# Supplementary material for: Stacking Model‐Based Classifiers for Dealing With Multiple Sets of Noisy Labels
Source: Biom J. 2025 Mar 12;67(2):e70042. doi: 10.1002/bimj.70042 (PMC11898607; doi:10.1002/bimj.70042)
Supplement: Supplementary file 1 — Supporting Information [file BIMJ-67-e70042-s001.zip › density-based-ensemble-model-main/competitors_real_data/netlab-master/netlab-master/help/rbfsetfw.htm]

Netlab Reference Manual rbfsetfw


# rbfsetfw

## Purpose

Set basis function widths of RBF.

## Synopsis

```
net = rbfsetfw(net, scale)
```

## Description

`net = rbfsetfw(net, scale)` sets the widths of
the basis functions of the
RBF network `net`.
If Gaussian basis functions are used, then the variances are set to
the largest squared distance between centres if `scale` is non-positive
and `scale` times the mean distance of each centre to its nearest
neighbour if `scale` is positive. Non-Gaussian basis functions do
not have a width.

## See Also

`rbftrain`, `rbfsetbf`, `gmmem`

---

**Pages:**
Index


---

Copyright (c) Ian T Nabney (1996-9)
